# Bottom and Top Internodes Subjected to Interactions with Genotype in Miscanthus: Impact of Biochemical Composition and Anatomy on Stem-Based Composites Mechanical Properties

**DOI:** 10.3390/polym17070966

**Published:** 2025-04-01

**Authors:** Maryse Brancourt-Hulmel, Stéphanie Arnoult, Jordi Girones, Sylvie Jaffuel, Thi To Loan Vo, Emilie Gineau, Gregory Mouille, Sophie Dubois, Patrick Navard

**Affiliations:** 1 BioEcoAgro Joint Research Unit-INRAE AgroImpact-Liège University-Lille University-Picardie Jules Verne University, Site of Estrées-Mons CS 50136, 80200 Péronne, France; 2 INRAE UE GCIE Picardie, Estrées-Mons, 80200 Péronne, France; stephanie.arnoult@inrae.fr; 3 Mines ParisTech, PSL–Research University, CEMEF-Centre de Mise en Forme des Matériaux, UMR CNRS 7635, CS 10207, Rue Claude Daunesse, 06904 Sophia Antipolis Cedex, Francepatrick.navard@minesparis.psl.eu (P.N.); 4 CIRAD, UMR AGAP, Avenue Agropolis, 34090 Montpellier, France; sylvie.jaffuel@cirad.fr; 5 Institut Jean-Pierre Bourgin, Université Paris-Saclay, INRAE, AgroParisTech, 78000 Versailles, Francegregory.mouille@inrae.fr (G.M.); 6 AnaStats Scop ARL, Les Vigneaux, 37220 Rilly sur Vienne, France; sophie.dubois@anastats.fr

**Keywords:** *Miscanthus* × *giganteus*, *Miscanthus sinensis*, biomaterial, composites, mechanical properties, internode

## Abstract

Miscanthus (Miscanthus Andersson) is a perennial grass for which biomaterials market has taken growing interest. Our objective was to evaluate the effect of stem internode position in *Miscanthus* × *giganteus* and *Miscanthus sinensis* and the impact of its anatomy and biochemical composition on internode-based composites’ mechanical properties. Stems’ bottom and top internodes were sampled for two genotypes of each species in two different years and separately added to a polypropylene matrix, and the mechanical properties of the internode-reinforced composites were measured. Before composite production, the internodes were extensively phenotyped for biochemical composition and anatomy. Stems’ bottom and top internode-based composites yielded different modulus (3203 and 2988 MPa, respectively), while tensile strength was similar (36.4 and 36.5 MPa, respectively). Significant genotype × internode interactions occurred for most variables, mainly due to differences among species, since both *Miscanthus sinensis* clones proved to be more stable than both *Miscanthus* × *giganteus* clones for modulus (4% and 10.2%, respectively). Regarding tensile strength, the species showed small but opposite differences between internodes. Tensile strength and modulus were rather close only in the top internodes, where good mechanical properties were associated with the lowest values of vascular bundles number and section area and highest parenchyma tissue, while opposite results were obtained in the bottom ones, only for tensile strength. *Miscanthus sinensis* species proved to be interesting for the stability improvement of composite mechanical properties. It appears essential for experimental purposes to stratify the sampling by internode in order to be representative of the whole stem.

## 1. Introduction

The search for eco-friendly materials from renewable resources has attracted great interest in recent decades, with a view to producing composites [1]. Composites correspond to a combination of two or more types of materials with superior properties than their individual components. A biocomposite is formed by using a natural material as reinforcement and a matrix created by a polymer from natural or synthetic sources [2].

Miscanthus, a crop that produces a high quantity of biomass with low nitrogen inputs [3] and pesticides [4], has received more attention in recent years for the development of such eco-friendly materials. This perennial crop is grown in Europe based on *Miscanthus* × *giganteus*, commonly known as giant miscanthus. It is a sterile interspecific hybrid whose genetic variability is known to be very limited since it comes from a single common ancestral clone [5,6]. Therefore, industrial use is restricted to one standard *Miscanthus* × *giganteus* clone [7], and such dependence on a single variety is risky at the slightest climatic or disease event. Another species of interest, *M. sinensis*, displays a very rich genetic pool in China [8] and can therefore contribute to widening miscanthus varietal offer while preserving the crop ecological interest and answering to wider quality requirements for different industrial uses.

The first composites reinforced with *Miscanthus* × *giganteus* were created in 2007 [9], where residue from the manufacture of horse bedding was used in the production of the poly-vinyl alcohol composites. The study showed that miscanthus allowed for improved flexural properties and can help reduce overall composite cost. Because of insufficient adhesion between the hydrophobic biopolymers and hydrophilic natural fibers, different techniques using a compatibilizing agent or corona discharge were then tested to improve miscanthus composites’ mechanical properties [10,11]. In 2016, Girones et al. [12] optimized a small-scale preparation procedure to differentiate genotypes in the reinforcing capacity for composites based on stem miscanthus fragments. The test evidenced a 16% difference for tensile strength and young modulus among the thirteen genotypes. Compared to neat-polypropylene, tensile strength increased significantly by 146%, and Young’s modulus by 255% on average. The minimum increases were obtained for a clone of the *Miscanthus sinensis* species (135% and 241% for tensile strength and Young’s modulus, respectively), while a clone from the *Miscanthus sacchariflorus* species displayed the highest increases (159% and 283%).

Miscanthus’ high productivity allows large annual harvests of lignocellulosic raw materials, with the proportion of cell-wall components varying according to species. Allison et al. [13] studied 244 accessions belonging to *M. sinensis*, *M. sacchariflorus*, and *M. × giganteus*. Compared with *M. sinensis*, *M.* × *giganteus* contains significantly more cellulose, which accounts for almost half of its cell wall (47% on average) and more lignin (acid detergent lignin = 11.8% on average) and less hemicelluloses (29.4%). A comparison of various preparations from miscanthus stalks has made it possible to identify the compositional parameters influencing the mechanical properties of composites [14,15]. Chupin et al. [14] studied in *Miscanthus* × *giganteus* the thermal properties of stem pieces carefully selected at the base and top of the whole stem and from the outer to inner position across the stem. In both genotypes studied, they found more hemicelluloses and less lignin in the upper part of stems and a significant effect of the hemicelluloses content in different radial layers of the stem but found no correlation with the thermal properties.

Few studies related the mechanical performances of miscanthus composites with both biochemical compositions and histological structure of the stems used as reinforcement. In a study comparing miscanthus to maize, the stem anatomy of the six miscanthus genotypes studied was characterized by a thick rind and a few dense pith bundles, while the stem biochemistry displayed high cell-wall, lignin, and cellulose concentrations [16]. These biochemical concentrations and stem-anatomy characteristics were positively correlated with strong mechanical properties in comparison to maize. However, no biochemical variables were considered. It can be noted that Kaack et al. [17] earlier explained the modulus elasticity of miscanthus by variables related to the anatomy and chemistry of the stems. However, they did not study stem-based composites but the stem characteristic itself in terms of modulus elasticity: they found that cellulose may be considered the most important variable compared to lignin because cellulose has the highest regression coefficients and interacts with two anatomical structures, area of parenchyma and area of vascular bundles, whereas lignin interacts with the area of the outer ring only, with a lower coefficient. In addition, other studies have also explored the strength properties of miscanthus stems [18,19], but no anatomical or biochemical variables were accounted for.

In order to widen the varietal offer of miscanthus and adapt the crop to new industrial applications, it is important to evaluate the genotype effect on mechanical properties and identify the biochemical traits and stem anatomy that may be addressed in breeding programs for improving the crop to utilize it in the composite components. Previous studies showed the influence of the miscanthus genotype on biochemical traits and stem anatomy. The impact of location on the stem has been studied with regard to the mechanical properties of composites, but has been concentrated on a single species, namely *M.* × *giganteus*. Accordingly, we hypothesized the existence of an interaction between genotype and stem internode on biochemical traits and internode anatomy, which can impact the mechanical properties of miscanthus composites. This would compromise the stability of the composite’s quality. Our main objective was to evaluate in miscanthus genotypes the effect of stem internode and its biochemical composition and anatomy on mechanical properties of internode-based composites, with particular attention being paid to genotype x internode interactions, as the literature reports no previous papers on this topic. Thus, analyses in biochemical composition and stem anatomy were extensively carried out in two different stem internodes of four miscanthus genotypes belonging to two different species, *M.* × *giganteus* and *M. sinensis*. Bottom (bot) and top (top) internodes were sampled for each genotype in two different years and added to a polypropylene matrix, and the mechanical properties of the internode-reinforced composites were measured.

## 2. Materials and Methods

### 2.1. Experimental Site and Climatic Conditions

**Figure 1:**
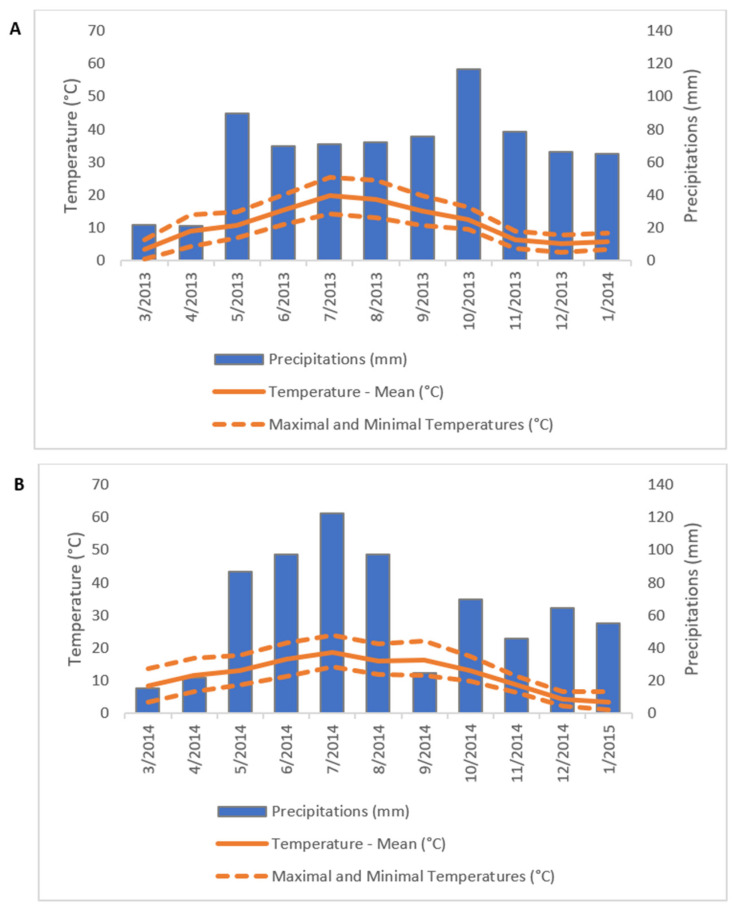
Rainfall and temperatures recorded during the two growing seasons of experiment, (**A**) 2013–2014 and (**B**) 2014–2015.

The experimental site was in the North of France (49°53’ N, 3°00’ E) at the INRAE experimental unit in Estrées-Mons. The climate in this French area corresponds to a degraded oceanic climate of the central and northern plains ([20]) and is characterized by a mean annual temperature of 11 °C and a mean annual precipitation below 700 mm over the 2007–2017 period, with generally low precipitation in summer. The precipitation and temperature data were collected throughout the experiment by a local meteorological station 1 km away from the trial. Monitoring meteorological data were downloaded from the INRAE CLIMATIK platform (https://agroclim.inrae.fr/climatik/, accessed on 13 December 2024) managed by the AgroClim laboratory of Avignon, France [21]. The ombrothermal diagrams in Figure 1 show the differences in the distribution of precipitation during both studied seasons. The two years studied (2013 and 2014) were intended to reflect this French region’s climate. Mean annual temperatures for both seasons matched the average of the climate, 10.0 and 11.5 °C, respectively, while annual precipitation was slightly higher, 738 and 755 mm, respectively, due to higher precipitation in summer for both years. In comparison to the first year (Figure 1A), precipitation amounts were higher between the months of June and August in the second year (Figure 1B), which corresponded to the flowering period. By contrast, these amounts were smaller in the months of September, October, and November of this second year.

### 2.2. Plant Material

Four contrasted miscanthus genotypes were cultivated at INRAE Estrées-Mons (49°53’ N, 3°00’ E) in a randomized complete block design with three replications.

Two genotypes were identified as *M.* × *giganteus* interspecific hybrids: a biomass clone (coded GIG_B); and an ornamental variety, Floridulus (FLO). Two other genotypes belonged to *M. sinensis* species: a biomass tetraploid Goliath variety (GOL) and a diploid ornamental Malepartus variety (MAL). The biomass clones (GIG_B and GOL) were provided by ADAS (Helsby, UK) and the Nordic biomass company (Hjørring, Denmark), respectively. Finally, both remaining ones (FLO and MAL) were acquired from the Chombard gardening nursery (Hombleux, France). Table 1 displays a morphological and phenological description of the genotypes studied. In addition to having a late heading date, both *M.* × *giganteus* clones (GIG_B and FLO) were characterized by higher canopy height, stem section, and biomass production than both *M. sinensis* (MAL and GOL). These genotypes were harvested in February 2014 and 2015, at the age of seven and eight years old, corresponding to an age when the plant has stabilized its production performance. As far as harvesting is concerned, winter harvesting was chosen, as it corresponds to farmers’ practices. All genotypes were harvested under the same maturity conditions, i.e., at over-maturity. It should be noted that autumn harvesting is not a good option for miscanthus, as it prevents the remobilization of nutrients from aboveground to belowground parts [3], which would compromise the sustainability of the crop.

### 2.3. Internode Sampling for Anatomical, Biochemical, and Mechanical Properties’ Analyses

In 2014, a plant sampling was carried out in the three blocks of the trial for anatomical analyses. Due to time-consuming and costly analyses, sampling was carried out in a single block (block 1) for both biochemical analyses and mechanical properties. In 2015, the sampling procedure was the same except for the biochemical analyses that were carried out in all blocks.

In each plot composed of 24 plants, 10 to 20 plants were harvested: (i) 10 plants were harvested, from which 5 to 15 highest stems per plant were sampled in order to carry out biochemical and anatomical analyses; and (ii) 15 to 20 plants were harvested, from which the 10 to 20 highest stems per plant were sampled for mechanical properties analyses.

For each stem, two internode ranks were sampled: the second internode from the bottom of the stem (bot) and the third from the top of the stem (top). These two contrasting internode levels were chosen in order to detect a gradient in the stem. Each internode was cleaned of leaves and sheaths. Sampled stem heights and sampled internode lengths were measured (Table 1).

### 2.4. Anatomical Analyses of Internodes

A 1 cm long segment was cut in the median part of each genotype internode sampled, thereafter fine cut. It was fixed in a buffer consisting of 100 mM phosphate (pH 7.2), with 1% (*v*/*v*) glutaraldehyde and 2% (*v*/*v*) (*w*/*v*) caffeine for 48 h at room temperature and then stored in 60% ethanol. The stem segments were then cut into thin 90 mm slices using a Vibratome Microm HM 650 V (Thermo Fisher Scientific, Bourgoin-Jallieu, France), and the slices were then stained overnight using a 1/7 diluted solution of Safranin and Alcian blue [22]. The staining solution was composed of 14 mL Alcian blue (0.5% in ethanol), 2 mL Safranin O (1% in water), 1 mL acetic acid, 30 mL glycerin, and 19.5 mL distilled water. After staining, sections were rinsed twice for 5 min with distilled water and mounted on glass slides in glycerol (50/50). Fasga staining was used to color cellulosic tissues blue and non-cellulosic tissues (essentially lignified) red. The prepared glass slides were then scanned with a Nanozoomer Hamamatsu and converted into high-resolution images. The resulting images were analyzed with the open-source ImageJ Version 1.43 freeware (https://imagej.net/ij/download.html, accessed on 7 November 2023) and dedicated script to quantify the following 8 traits: The internode section area (IN_Area, mm^2^) and the external area, which corresponded to the stem section rind fraction, were expressed in percentage of the internode section area (Area_Zext). In the external area, the percentage of sclerenchyma tissue was quantified in percentage of external area (Scl_Zext). In the internal area, the percentage of parenchyma tissue (Parenchym), the percentage of empty space (EmptySpace_Zint), and the percentage of blue corresponding to less lignified cell wall (Blue_Zint) were quantified and expressed in percentage of internal area. The density of vascular bundles (VB_density, number/mm^2^) was also quantified in the internal area. The number of vascular bundles in the internal and central areas (VB_number) was also determined as visual differences were detected in miscanthus. The external area (Zext) and the internal area (Zint) were delimited visually based on the anatomical difference between the two areas and, in particular, vascular bundles sizes and positions.

### 2.5. Preparation of Plant Samples for Biochemical Analyses and Composites Realization

After being mildly dried, 50 g miscanthus samples of each genotype internode were grinded in a Hellweg M50 granulator equipped with a 2.5 mm sieve. Fragments sizes were further reduced by means of a coffee mill (Carrefour home, Massy, France) operated for 1 min in sequences of 10 s. In order to ensure homogeneous fragment size, stem fragments were subsequently sieved in a Retsch AS200 Digit shaker (Retsch, Haan, Germany). Sieving was conducted on 20 g samples, with the shaker operating at a 40 mm amplitude (2 mm/g) for 5 min. Sieves with open pore sizes of 1000, 600, 400, 300, 200, and 100 μm were used. The fraction collected in the 100 μm sieve was selected as the reinforcing element, whilst larger fragments were submitted to successive grinding/sieving processes until enough fragments of the selected size were collected. Prior to compounding, miscanthus fragments were dried overnight in an air-circulating oven operating at 60 °C.

### 2.6. Biochemical Analyses of Internodes

Approximately 1 g of each previous plant powder sample of each genotype internode was used for their biochemical characterizations. Before exhaustive water, ethanol was extracted in a Soxhlet apparatus. The recovered extractive-free samples, corresponding to cell walls, were dried at 50 °C before their compositional analyses. The lignin content (ABL) was measured using the acetyl bromide lignin method according to Sibout et al. [23]. The % of p-hydroxyphenyl (H), guaiacyl (G), and syringyl (S) units in lignin was also determined by thioacidolysis, as described in Mechin et al. [24].

The hemicellulose and cellulose levels, as well as the hemicellulosic neutral sugars, were measured as described in Chupin et al. [14]. The hemicellulosic neutral sugars that were characterized were the following: arabinose (Arabinose), galactose (Galactose), glucose (GluHem), and xylose (XylHem). The amounts of hemicelluloses (TotHem) and glucose from cellulose (GluCell) were also measured as described in Chupin et al. [14].

The main phenolic acids, ester-linked p-coumaric acid (CA) and ferulic acid (FA), were measured by mild alkaline hydrolysis, followed by solid-phase extraction and then HPLC analyses according to Ho-Yue-Kuang et al. [25].

### 2.7. Mechanical Properties of Internodes-Based Composites

#### 2.7.1. Preparation of the Polymeric Matrix

A homopolymer polypropylene, Addilene (Arkema, Nanterre, France) with a MVI of 70 g/10 min), was used as polymeric matrix. Maleic anhydride-grafted polypropylene (MAPP), commercialized under the name of G-3015 (Eastman, Puteaux, France), was used as coupling agent in a proportion of 5% *w*/*w* on a dry miscanthus fragment basis. Prior to use, coupling agent was kept in an air-circulating oven overnight at 60 °C in order to activate the maleic anhydride groups.

#### 2.7.2. Composite Preparation

Composite blends comprising 30% (*w*/*w*) miscanthus powder samples of each genotype internode, polymer, and coupling agent were prepared with a HAAKE Rheomix intensive kinetic mixer and at 180 °C, with a mixing process lasting 9 min. After compounding, the composites were granulated in a blade mill and kept in an oven at 80 °C before being injection-molded in a Haake Minijet-II (Thermo Fisher Scientific, Bourgoin-Jallieu, France) using a steel mold (ISO 527-2 [26]).

For each sampled internode of each miscanthus genotype, 40 g of polypropylene-based composites reinforced with 30% of miscanthus was prepared.

#### 2.7.3. Mechanical Characterization

Composite specimens were left to rest in a conditioned room at 25 °C and 50% humidity for at least 3 days before testing. Tensile tests were carried out in a Zwicki Z2.5 tensile testing machine (Zwick-Roell, Ars-Laquenexy, France) displaying a force cell of 2.5 kN, operating at 0.02 mm/s (1.2 mm/min), with a 55 mm gap between grips and 2 kN pre-tension (ASTM D618-21 [27]). Due to the small sizes of test bars, no extensometer was used to monitor the strain of the composite specimens in order to avoid a potential disruptive effect. It is important to note that even in the case of biases, all genotypes were compared under the same conditions, where biases were equivalent between genotypes and minimized as much as possible. Young’s modulus was determined from the secant of the stress–strain curve at 0.05–0.25% deformation. Each reported result for tensile strength and Young’s modulus corresponds to the mean value of ten tests.

### 2.8. Data Analysis

Statistical analyses were performed using R software (3.6.1 version).

For biochemical and anatomical data, the experimental design was analyzed using a linear mixed model to test the effects of year, genotype, internode position, and the corresponding interactions:

Y_ijkl_ = µ + α_i_ + β_j_ + γ_k_ + αβ_ij_ + αγ_ik_ + βγ_jk_ + αβγ_ijk_ + B_l_ + ε_ijkl_

where Y_ijkl_ is the value of the trait for year i, genotype j, internode position k, and block l; µ corresponds to the overall mean; α_i_ is the fixed effect of year i; β_j_ is the fixed effect of genotype j; γ_k_ is the fixed effect of internode position k; αβ_ij_ is the fixed interaction between year i and genotype j; αγ_ik_ is the fixed interaction between year i and internode position k; βγ_jk_ is the fixed interaction between genotype j and internode position k; αβγ_ijk_ is the fixed interaction between year i, genotype j, and internode position k; B_l_ is the random effect of block l; and ε_ijkl_ is the residual error for year i, genotype j, internode position k, and block l.

For mechanical properties, a linear model was used where the block in the previous model was replaced by a technical replication declared as a fixed effect.

As the models’ residuals were not normally distributed after parametric tests, nonparametric tests were performed. Aovperm() function of the {permuco} package was used [28] to provide *p*-values based on permutation tests for the ANOVA model. Permutation tests were performed using freedman_lane to handle nuisance variables and 5000 permutations.

Pearson correlation coefficients between the anatomical and biochemical variables that exhibited the most significant internode x genotype interactions in the previous ANOVA (corresponding to *p*-values < 0.001), as well as the two mechanical properties, were performed by using the cor() function. 

Correlation matrix containing the previous anatomical and biochemical variables was analyzed according to principal component analysis (PCA). These variables were considered active variables, while both mechanical properties were used as supplementary or illustrative variables. The interpretation of the correlation matrix was conducted with the analysis of a plot displaying correlations between each variable and each principal component in Cartesian diagrams. Because the analysis was carried out on the correlation matrix, the principal component loadings are standardized, and the magnitude and sign of the loadings essentially reflect the correlation of the variable to the principal component. Variables with loadings around 0 are not correlated to the principal component. The sign of the loading indicates whether the variable is positively or negatively correlated to what the principal component is summarizing. PCA analyses were performed by using the {factoextra} package. The plot of individuals was displayed by using the fviz_pca_ind() function and the plot of variables by the fviz_pca_var() function.

## 3. Results

### 3.1. Internodes Displayed Great Effects in Interaction with Genotype and Year for Most Variables, Except for Tensile Strength

The four genotypes displayed differences in length of stems, as well as internodes (Table 1). Both *M.* × *giganteus* clones, GIG_B and FLO, showed higher stems than both *M. sinensis*, MAL and GOL: they reached 2.7 m and 1.6 m over the two years, on average, respectively. The length was stable for both *M. sinensis* in contrast to that of both *M.* × *giganteus*. Moreover, in contrast to MAL and GOL genotypes, the bottom internodes of GIG_B, and FLO to a lesser extent, were longer than the top internodes (Table 1). The mean differences between the two internodes for both years reached 11.7 cm, while both *M. sinensis* showed top internodes that were somewhat longer than the bottom ones. MAL even showed the greatest stability of internode length between bottoms and tops.

The internode main effect displayed the highest F values in the ANOVA table (Table 2) for most of the traits, except for tensile strength. The anatomical variables studied were significantly and mainly influenced by the internode position in the stem, except for the number of vascular bundles in the internal and central areas (VB_number), as this number was mainly influenced by year and genotype, respectively.

Among the anatomical traits that were highly influenced by internode position, the bottom internodes (bot) were characterized by a higher internode section area (IN_Area, with 56.6 and 29.3 mm^2^ for bottom and top, respectively), a higher empty space in the internal area (EmptySpace_Zint, recording 20.6 and 3.5%, respectively), a greater external area corresponding to rind fraction (Area_Zext with 25.9 and 16.5% of section area, respectively), and a higher sclerenchyma percentage in the external area (Scl_Zext with 55.8 and 45.3%, respectively) than the top internodes (Table 3). In contrast, they displayed a lower percentage of parenchyma tissue in the internal area (Parenchym) and a lower density of vascular bundles in the internal area (VB_density) than top internodes with 45.9% versus 76.6 and 2.8 nb/m^2^ versus 3.8, respectively. Among the most significant biochemical traits related to stem composition, internodes differed in lignin content and structure. Higher lignin contents were observed for bottom internodes than for top internodes (ABL with 24.8 and 22.1% of cell wall for bottom and top, respectively). About the structure, they showed a lower proportion of p-hydroxyphenyl (H) and guaiacyl (G) subunits, with 1.7 versus 3.1% and 57.9 versus 59.8% for bottom and top, respectively, but a higher proportion of syringyl subunit (S), with 40.3 and 37.1%, respectively. In addition, bottom internodes showed lower contents of ferulic acid (FA) than top internodes (3.8 mg/g versus 5.0, respectively) but higher p-coumaric acid (CA with 18.7 mg/g versus 17.2). They also showed a lower total of hemicelluloses (TotHem with 173.6 mg/g versus 227.4) for which arabinose and xylose constituted the most significant variables, with 17.1 mg/g versus 23.8, and 148.3 mg/g versus 193.4, respectively (Table 3). 

**Figure 2:**
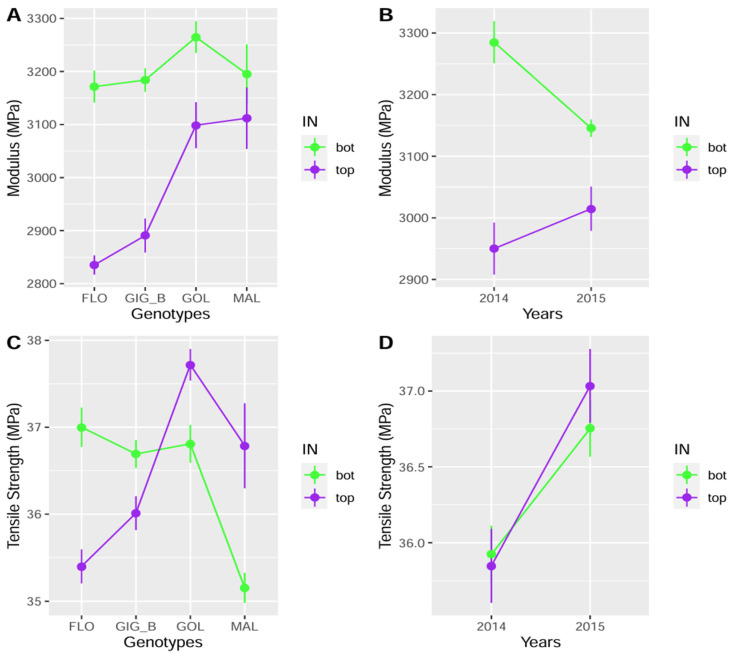
Mechanical properties of internode-based composites for the four genotypes (modulus in (**A**) and tensile strength in (**C**)) in 2014 and 2015 (modulus in (**B**) and tensile strength in (**D**)).

This high internode effect was accompanied by significant interaction effects with the genotype for several variables. Consequently, genotypes cannot be compared by internode, but comparisons must focus on the differences between internodes among genotypes. It was noticeable for both mechanical properties and a rather high number of biochemical and anatomical traits. For modulus, the MAL genotype displayed the lowest difference between its top and bottom internodes, reaching 83.3 MPa, which corresponded to 2.6% of its average modulus (3150 MPa; see Figure 2A). In contrast, the FLO genotype displayed the highest difference of 420 MPa, which corresponded to 14.0% of its average (3000 MPa). Interestingly, both *M. sinensis* showed a lower difference between their top and bottom internodes (4% on average), compared to both genotypes of *M.* × *giganteus* (10.2% on average). As a result, the *M. sinensis* clones appeared more stable. With regard to tensile strength (Figure 2C), all genotypes showed small differences between internodes, which were similar in magnitude, but *M. sinensis* (MAL and GOL) and *M.* × *giganteus* (FLO and GIG_B) showed opposite results (3.1% and −3.5%, respectively). Regarding the anatomical traits, the genotype x internode position interaction was significant and particularly high with regard to the F values for IN_Area, Parenchym, EmptySpace_Zint, VB_density, and VB_number (see F values in Table 2). Concerning the biochemical traits, the interaction between genotype and internode position was rather high for ABL, CA, FA, Rhamnose, G, S, GluHem, and Arabinose.

The internode also interacted with the year but for fewer variables than with genotype. Modulus was significantly impacted (Figure 2B), for which a much greater difference was observed between bottom and top internodes in 2014 in comparison to 2015, +335 MPa and +131 MPa, respectively: this corresponded to 10.7% and 4.2% of the respective averages (3118 MPa and 3081 MPa). For tensile strength, the differences were small between the two years (Figure 2D) and not significant (Table 2). Among the anatomical variables, Parenchym, EmptySpace_Zint, and VB_number were subjected to internode x year interactions, while CA, G, S, and GluHem were influenced among the biochemical traits.

Finally, a triple interaction between internode, genotype, and year was observed for both mechanical properties (Table 2). In the same table, such an interaction was also visible for anatomical traits, such as Area_Zext and VB_number, as well as biochemical traits, such as ABL and CA. Interestingly, only the VB_number anatomical trait and CA biochemical trait displayed significant effects for all interactions involving internodes (i.e., genotype x internode, year x internode, and year x genotype x internode), as well as moduli.

### 3.2. The Bottom and Top Internodes Showed Contrasted Correlations Between Tensile Strength and Some Anatomical Variables, While Stable Correlations Were Observed Between Years

A correlation analysis was carried out with variables exhibiting the most significant internode x genotype interactions in the previous ANOVA table (corresponding to *p*-values < 0.001). The bottom and top internodes were initially distinguished, and then the years.

**Figure 3:**
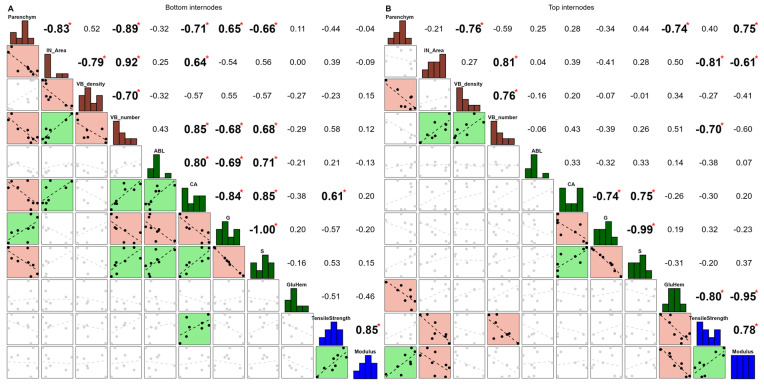
Pearson correlation analysis based on bottom (**A**) and top internodes (**B**) and carried out with the variables exhibiting the most significant internode x genotype interactions in Table 2 (*p*-values < 0.001). *p*-values significance: ‘*’ < 0.05. On the diagonal, anatomical variables appear in brown and biochemical in green. Mechanical properties are colored blue.

There was a rather strong correlation between the two mechanical properties regardless of the internode (0.85 and 0.78 for bottom and top internodes, Figure 3A and B, respectively). Regarding the bottom internodes, strong correlations were noticeable between and within each group of anatomical and biochemical variables, except for GluHem (Figure 3A). CA was positively correlated with modulus (0.61). In the top internodes, several correlations were significant with the anatomical variables (Figure 3B).

Focusing on the comparison of both internodes, it is interesting to note some sign changes in the correlations between the variables. This was the case of the correlation between Parenchym and VB density (0.52 and −0.76 for bottom and top, respectively), as well as IN Area and VB_density (−0.79 and 0.27, respectively). Parenchym and IN_Area also displayed changes with tensile strength (−0.44 and 0.40, respectively; 0.39 and −0.81, respectively). There was also an inversion of the correlation between VB density and number (−0.70 and 0.76, respectively), as well as inversion between VB_number and tensile strength (0.58 and −0.70, respectively).

**Figure 4:**
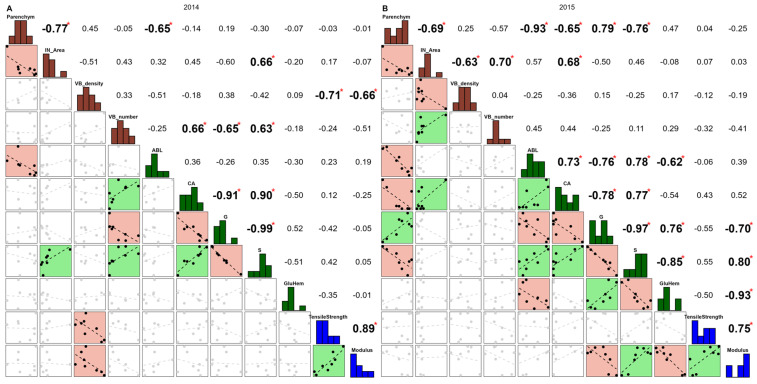
Pearson correlation analysis based on 2014 (**A**) and 2015 (**B**) and carried out with the variables exhibiting the most significant internode x genotype interactions in Table 2 (*p*-values < 0.001). *p*-values significance: ‘*’ < 0.05. On the diagonal, anatomical variables appear in brown and biochemical in green. Mechanical properties are colored blue.

Comparing the two years, a greater number of correlations were significant in 2015 (Figure 4B) compared to 2014 (Figure 4A). With the exception of VB_number and VB_density, most correlations within each group of biochemical and anatomical variables were significant in 2015 (Figure 4B). Accordingly, most biochemical variables impacted the modulus: positively for CA, and S (0.52, and 0.80, respectively); and negatively for G and GluHem (−0.70 and −0.93). In 2014 (Figure 4A), VB_density and VB_number variables negatively impacted the modulus (−0.66 and −0.51, respectively), as well as VB_density for tensile strength, with a correlation reaching −0.71.

Unlike internodes, great stability was observed between years among the correlations. It seemed that the interactions observed with the years in the ANOVA did not impact the sign of these correlations. It constituted the main difference between the year and internode effects as the two positions of internodes generated inversions in the correlations between the mechanical properties and some variables among the anatomical and biochemical traits.

### 3.3. A Principal Component Analysis Based on Variables Showing the Most Significant Internode x Genotype Interactions Clearly Separated the Bottom and Top Internodes

**Figure 5:**
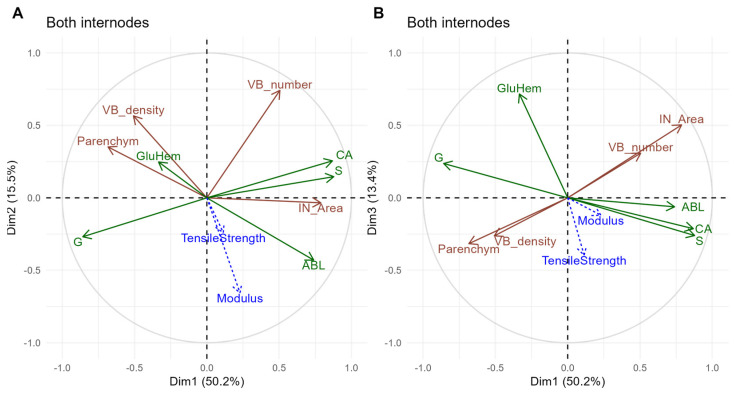
Principal component analysis of both internodes based on the anatomical and biochemical variables exhibiting the most significant internode x genotype interactions in the ANOVA Table 2 (*p*-values < 0.001). Plot of correlations between the variables on the first two components in (**A**) and on the first and third components in (**B**). Anatomical variables appear in brown and biochemical in green. Mechanical properties are colored blue.

Correlations between anatomical and biochemical variables were analyzed according to a principal component analysis (Figure 5). The variables exhibiting the most significant internode x genotype interactions in the initial ANOVA table were accounted for (corresponding to *p*-values < 0.001). The first component captured 50.2% of the variation in the correlation matrix (Plots A and B). In the plot of variables (biochemical variables in green, and anatomical in brown), this component indicated that as S (loading + 0.88), CA (loading + 0.87), IN_Area (loading + 0.79), and ABL (loading + 0.74) increased, G (loading − 0.86) and Parenchym (loading − 0.68) decreased (Plot A).

The second component accounted for 15.5% of the variation in the correlation matrix (Plot A). This component indicated that, as VB number (loading + 0.74) and density (loading + 0.57) increased, ABL (loading − 0.42) and G (loading − 0.27) decreased.

**Figure 6:**
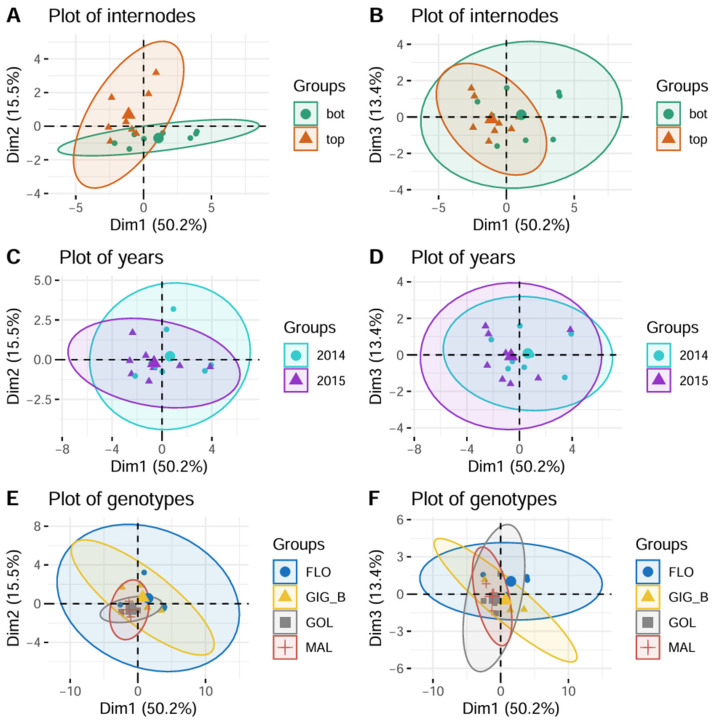
PCA plots of individuals are differentiated according to internodes (on the first two components and on the first and third components, (**A**) and (**B**), respectively), years ((**C**) and (**D**), respectively), and genotypes ((**E**) and (**F**), respectively).

Interestingly, the plot of individuals (i.e., combinations of genotypes, internodes, and years) along the first component highlighted combinations related to the bottom internodes (see Plot A differentiating the internodes, Figure 6), while the second and third components depicted combinations related to the top ones (Plots A and B). Unlike the internodes, which showed a clear separation, the two years overlapped rather well (plots C and D differentiating the years). In the plots where the individuals were differentiated according to the genotypes (Plots E and F), the points related to both *M. sinensis* genotypes (MAL and GOL) were grouped on the first two components (Plot E), unlike the two *M.* × *giganteus*, which showed a much greater dispersion, the FLO genotype showing the greatest (Plot F): differences among the internodes were much smaller for the variables under consideration in the *M. sinensis* than in the *M.* × *giganteus*.

### 3.4. A Good Performance in Mechanical Properties Was Associated with the Highest Parenchym Values and Lowest VB_Number and IN_Area in the Top Internodes

**Figure 7:**
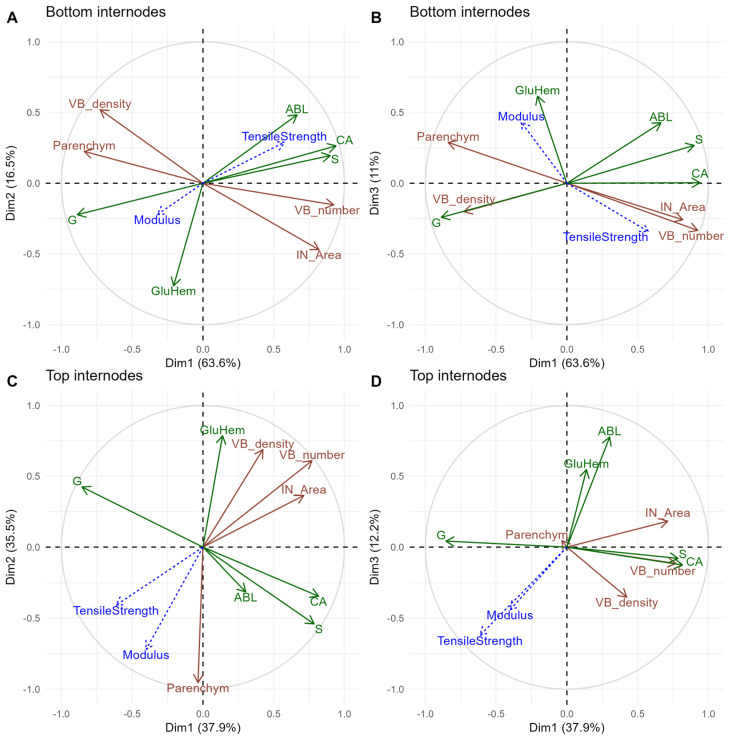
Principal component analysis by internode based on the same variables as in Figure 5. Plot of correlations between the variables on the first two components in (**A**) and on the first and third components in (**B**) for the bottom internodes. Plot of individuals on the first two components in (**C**) and on the first and third components in (**D**) for the top internodes. Anatomical variables appear in brown and biochemical in green. Mechanical properties are colored blue.

The previous graphs show rather distinct groupings of individuals according to internodes, so a deeper analysis was carried out for internodes (Figure 7). In the plot related to the bottom internodes, the first component accounted for a very high value of 63.6% of the variation in the correlation matrix between anatomical and biochemical variables (Plot A of Figure 7). The first axis indicated that the more the CA (loading + 0.94), S (loading + 0.90), IN_Area (loading + 0.82), and VB number (loading + 0.92), as well as ABL but to a lesser extent (loading 0.66), increased, the more G (loading − 0.89) and Parenchym (loading − 0.84) decreased. The second component captured 16.5% of the variation in the correlation matrix (Plot A of Figure 7). The second axis indicated an opposition between ABL and GluHem, as ABL was positioned at the positive end (loading + 0.48), and GluHem at the negative end (loading − 0.73).

Of the two mechanical properties, only tensile strength was fairly well explained on this plot related to the bottom internodes: positioned on the positive end of the first axis, high levels of tensile strength were associated with the highest values of CA, S, ABL, VB_number, and IN_Area, as well as with the lowest values of G and Parenchym.

In the plot related to the top internodes (Plot C of Figure 7), tensile strength and modulus were rather close and therefore associated with similar biochemical- and anatomical-trait levels. The first axis captured 37.9% of the variation in the correlation matrix, which was rather low compared to previous bottom internodes. This axis showed an opposition between CA (loading + 0.82), S (loading + 0.79), VB number (loading + 0.77), and IN_Area (loading + 0.71), all located at the positive end of the axis, and G, which was positioned at the negative end (loading − 0.86). The second axis accounted for a similar proportion of the variation in the correlation matrix compared to the first axis (loading 35.5%). This component indicated that as GluHem increased (loading + 0.79), Parenchym decreased (loading − 0.95).

As for the bottom internodes, a good tensile strength was associated with high CA and S values. However, the main difference appeared with Parenchym, VB_number, and IN_Area: in these top internodes, a good performance in tensile strength, as well as modulus, was associated with the highest values of Parenchym and the lowest VB_number and IN_Area. This opposition between bottom and top internodes explained why the internode exhibited no significant effect for tensile strength in the initial ANOVA.

## 4. Discussion

Until now, few studies have dealt with the link between stem characteristics and mechanical properties of plant stem-based composites. Therefore, this study brings to light new knowledge of miscanthus anatomical and biochemical stem properties in relation to stem-based composites mechanical properties, with a particular focus on the position in the stem. Our main hypothesis was that the localization in the stem, i.e., internode position, has an impact on biochemical and anatomical characteristics, which in turn impact mechanical properties. We also predicted variations among genotypes.

Our study showed differences in length in the stems, as well as internodes, among the genotypes. A high internode effect was highlighted for all traits, and this effect was accompanied by many significant interaction effects with the genotype and year. Interestingly, the VB_number anatomical trait and CA biochemical trait displayed significant effects for all interactions involving internodes, as well as modulus. In addition, some anatomical and biochemical traits were also highlighted regarding internode x genotype interactions. Accounting for the anatomical and biochemical variables exhibiting the most significant internode x genotype interactions in the ANOVA, PCA analyses separated by internode position highlighted contrasting relationships between mechanical properties and some of these variables. In the bottom and top internodes, a good tensile strength is associated with high CA and S values. However, the main differences between the bottom and top internodes appeared with Parenchym, VB_number, and IN_Area: in the bottom internodes, a good performance in tensile strength was associated with the highest values of VB_number and IN_Area, and lowest Parenchym, while the opposite results were obtained in the top. Tensile strength and modulus were correlated only in top internodes. This opposition between bottom and top internodes explained why the internode showed no significant effect for tensile strength in the initial ANOVA. Despite significant interactions with the year, a great stability was observed between years among all significant correlations between and within each group of anatomical and biochemical variables, as well as correlations between these variables and mechanical properties.

Below, we first discuss the differences between bottom and top internodes for anatomical and biochemical characteristics and their correlations with mechanical properties, and second, we focus on the interaction effects of internode with genotype and year. Finally, we discuss the need for recommendations on stem samplings for future studies regarding stem-based composites’ mechanical properties.

### 4.1. Bottom and Top Stem Internodes Displayed Some Contrasted Anatomical and Biochemical Characteristics, Which Impacted the Mechanical Properties of the Corresponding Internode-Based Composites

This study showed that miscanthus internodes differed for several anatomical and biochemical characteristics. In comparison to top internodes, bottom internodes displayed higher section area and lignin content (with lower proportions of G and H but higher S lignin subunits), with more rind fraction encompassing the epidermis (Area Zext), as well as higher empty space in internal area (IN_area) and sclerenchyma percentage in the rind (Scl Zext). In contrast, they presented a lower percentage of parenchyma tissue in internal area (Parenchym) and a lower density of vascular bundles in pith (VB_density). Moreover, less hemicelluloses were also deposited in the older internodes compared to the younger (TotHem). Kaack et al. [17] analyzed several genotypes of miscanthus belonging to several species, including *M. sinensis* and *M.* × *giganteus*. The concentration of cellulose varied significantly between genotypes; however, no significant differences were found between internodes. The concentration of lignin varied significantly between genotypes, decreasing significantly from the lower to the upper internodes. The thickness of the outer ring, number of vascular bundles, and area of vascular bundles also varied significantly between genotypes, and they decreased significantly from the lower to the upper internodes. The area of outer ring and area of parenchyma decreased significantly from the lower to the upper internodes and varied significantly between genotypes. Some of these features were also reported in other perennials. In tall fescue tissues (*Festuca arundinacea* Schreb.), Chen et al. [29] reported similar observations between older basal internodes and younger upper internodes, with a decrease in lignin content, sclerenchyma area, and wall thickness, as well as the same trends for lignin subunits and hemicelluloses. In another perennial crop, the study of alfalfa (*Medicago sativa* L.) also showed that younger stem internodes had lower cell-wall and Klason-lignin concentrations than older internodes [30]. In hemp, Beaugrand et al. [31] observed a significant decrease in lignin content and an increase in arabinose and galactose contents from the bottom to the top of the stems.

Accordingly, it was difficult to explain modulus and tensile strength by the anatomical and biochemical characteristics in a global principal component analysis gathering the two internodes. This was mostly due to the high effect of the internode, which masked the impact on both mechanical properties. In correlation matrices carried out separately by internode, it was indeed possible to relate mechanical properties to particular biochemical and anatomical traits and note the existence of contrasted correlations between mechanical properties and some of these traits among internodes. In the bottom internodes, a good performance in tensile strength was associated with the highest VB_number and IN_Area and lowest Parenchym values, while opposite results were obtained in the top ones. However, it was associated with high values of CA and S in both internodes. Such contrasting results may suggest that some other parameters are playing a role, masking or changing correlations. During the preparation of the composites, effects of temperature and pressure may impact the stability of the stem fractions, different in the top and in the bottom, potentially explaining these opposite correlations between top and bottom. Such an effect of temperature and pressure on internodes’ stability has to be further explored for composite valorization, at least in *Miscanthus* × *giganteus*.

In a study comparing maize and miscanthus and where bottom internodes were accounted for in miscanthus, cell-wall, lignin, and cellulose concentrations were positively correlated with rind fraction and pith bundle density and accounted for good mechanical properties [16]. Our study showed that particular attention should be paid to the internode position in the study of stem-based composites mechanical properties. We indeed explained the modulus and tensile strength of bottom and top internode composites by differentiated anatomical and biochemical characteristics.

### 4.2. Differences Among the Internodes Were Much Smaller in the M. sinensis than in the M. × giganteus for Most Variables, Which Involved More Stable Mechanical Properties

Along with this high internode effect, highly significant genotype x internode interactions were noticeable for many variables: both mechanical properties were impacted as well as a rather high number of biochemical and anatomical traits. For the composite modulus, the MAL genotype displayed the lowest difference between its bottom and top internodes (2.6% of its average modulus), while the FLO genotype displayed the highest difference (14.0%). The anatomical traits highly subjected to interaction effects concerned IN_Area, Parenchym, EmptySpace_Zint, VB_density, and VB_number, whereas biochemical traits were related to ABL, CA, FA, Rhamnose, G, S, GluHem, and Arabinose. The interaction was mainly due to a species effect: a principal component analysis based on all of these traits showed that the points related to the two genotypes of the *M. sinensis* species were grouped, unlike the genotypes of *M.* × *giganteus*, for which the FLO genotype displayed the highest dispersion in terms of biochemical and histological parameters.

It is interesting to note that focusing on the modulus elasticity of the stem, Kaack et al. [17] highlighted that *M.* × *giganteus* ranked among the best genotypes, displaying high stem rigidity, modulus of elasticity, and cellulose in interaction with area of vascular bundles. In our results, both VB_density and VB_number were differentially highlighted in the bottom and top internodes, but the modulus elasticity of the stem was not measured. It would have been interesting to relate this trait to the mechanical properties of the composites, and this variable needs to be explored in the future regarding composite valorization.

In consequence, our results provide new insights regarding such an interaction effect. In particular, *M. sinensis* species would be interesting, as it would help reduce the differences between internodes and therefore improve stem-based composites’ stability.

### 4.3. Insight for Stem-Based Composites Preparation in Miscanthus

This study pointed out novel phenotyping recommendations for future samplings. When studying the mechanical behavior of miscanthus stem fragments, it proves necessary to compare the results on specific internode positions and relate the mechanical behavior to the corresponding biochemical and histological parameters, as performed in the present study. According to Chupin et al. [14], the lack of traceability of the sample origin —such as top and bottom internodes—in composite preparation is probably one of the potential sources of the variability between published results, and this variability source is a difficulty for composite preparation at elevated temperatures.

To simplify the sampling process while reducing such stem heterogeneity, some studies of miscanthus composites were based only on bottom internodes. While it provided better correlations between biochemical and histological parameters and mechanical properties [16], it did not represent the whole stem due to the existence of genotype x internode interactions, as evidenced in the present study. In contrast to maize, where the elongated internode below the main cob is the most representative of stem internodes and where this internode sampling was used to perform histological experiments [32], it seems more complicated to reduce the analyses to a single internode in miscanthus.

Other studies have separated panicles from stems and leaves because they react differently to temperature, with the stem showing optimal thermal stability at low-temperatures, while the panicle excels in high-temperature conditions [33]. Leaves could be another source of heterogeneity; this is why each internode was cleaned of leaves and sheaths. However, it is important to note that the leaf/stem biomass ratio of a given plant is very low: from mid-May to panicle emergence in autumn, Zapater et al. [34] observed a decrease in this ratio from 3 to 0.2 and from 2.5 to 0.4 for *Miscanthus* × *giganteus* and *Miscanthus sinensis*, respectively. At winter harvest, few leaves remain as this ratio even dropped below 0.1 and 0.3, respectively [35]. In contrast, Mangold et al. [36] documented leaf and stem ratios in four genotypes harvested on three dates between mid-September and mid-October but did not study winter harvest, as their objective was to focus on biogas production and not on composites. As the crop is generally harvested in late winter or early spring to allow full nutrient recycling and low crop moisture, leaf removal could therefore be neglected in the process of future samplings.

Consequently, our results show that, at the experimental level, the evaluation of miscanthus for stem-based composites must be carried out with care due to the existence of multiple genotype x internode interactions. Indeed, it seems essential to implement a stratified sampling design in which different internodes of a given stem are sampled, grinded, and carefully mixed in order to represent the whole stem, thus providing access to the quality stability of stem-based composites for a given genotype.

## 5. Conclusions

This study compared two clones of *M. sinensis* with two clones of *M.* × *giganteus* regarding cell-wall composition and stem anatomy for conversion into polymer composites. The four clones showed differences in length in stems, as well as bottom and top internodes. Stems’ bottom and top internode-based composites yielded different moduli (3203 and 2988 MPa, respectively), while tensile strength was similar (36.4 and 36.5 MPa, respectively). The study highlighted internode effects regarding anatomical and biochemical traits for which bottom internodes highly differed from top internodes for many variables. Polypropylene-based composites reinforced by bottom and top internodes were also impacted in turn, regarding the corresponding mechanical properties. Along with this high internode effect, highly significant genotype x internode interactions were noticeable and mainly due to a species interaction effect for which both *M. sinensis* clones appeared more stable than both *M.* × *giganteus* clones regarding modulus. Both *M. sinensis* indeed showed a lower difference between their top and bottom internodes (4% on average), compared to both genotypes of *M.* × *giganteus* (10.2% on average). With regard to tensile strength, all genotypes showed small differences between internodes, which were similar in magnitude, but *M. sinensis* (MAL and GOL) and *M.* × *giganteus* (FLO and GIG_B) showed opposite results (3.1% and −3.5%, respectively). Therefore, *M. sinensis* species should be interesting for the stability improvement of composite mechanical properties. In addition, this study showed that particular attention should be paid to the internode position when examining stem-based composites mechanical properties. It appears essential for experimental purposes to stratify the sampling of internodes in order to obtain a more representative sampling of the whole stem, thus providing access to the quality stability of stem-based composites.

In terms of future research prospects, the effects of temperature and pressure may have an impact on the stability of internode stem fractions during composite preparation, particularly in *Miscanthus* × *giganteus*, which showed the greatest differences in composite mechanical properties between internodes. This effect of temperature and pressure on internode stability needs to be studied in greater detail in the future for the valorization of composites. On the plant side, the effect of the environment may be another source of variation, involving interactions with genotype and internode. A multi-location trial focusing on the variables highlighted in this article would enable us to assess the effect of the environment on the mechanical properties of internode-based composites.

## Figures and Tables

**Table 1:** Morphological and phenological description of the four genotypes studied, FLO, GIG_B, GOL, and MAL, with the measures of the stems and internodes sampled.

Trait		Canopy Height at the End of Season (m)	Stem Section (mm)	Heading Date	Biomass Production (t DM/ha)		Mean Height of the Stems Sampled (m)		Mean Length of the Internodes Sampled (cm)	
Year		Mean of the years 2011 to 2013 (Fourth to sixth years of cultivation)	Mean of the years 2011 to 2013 (Fourth to sixth years of cultivation)	2013 (sixth year of cultivation)	2014	2015	2014	2015	2014	2015
Genotype	FLO	2.9	10	No heading appearance	28.4	40.6	2.4 ± 0.01	3.1 ± 0.2	Bot: 29.5 ± 4.4 Top: 19.7 ± 2.0	Bot 15.8 ± 5.3 Top: 12.4 ± 2.1
	GIG_B	2.8	10	No heading appearance	26.1	34.2	2.4 ± 0.1	2.9 ± 0.2	Bot: 25.9 ± 2.4 Top: 12.3 ± 0.9	Bot: 18.3 ± 3.1 Top: 10.7 ± 1.2
	GOL	1.9	7	29/08/2012	22.8	20.5	1.9 ± 0.3	1.8 ± 0.1	Bot: 9.5 ± 2.8 Top: 14.9 ± 0.7	Bot: 9.3 ± 1.2 Top: 15.1 ± 2.2
	MAL	1.4	6	20/08/2012	12.1	10.9	1.3 ± 0.05	1.4 ± 0.06	Bot: 7.8 ± 1.0 Top: 11.8 ± 0.4	Bot: 9.6 ± 3.7 Top: 11.6 ± 3.1

**Table 2:** F values and degrees of freedom (df) corresponding to ANOVA for (a) mechanical properties of internode-based biocomposite variables, (b) histological variables, and (c) biochemical variables, measured on 4 genotypes (GIG_B, GOL, FLO, and MAL), for 2 internode positions (bottom and top), in 2 years (2014 and 2015). Main effects highlighted in blue for the most significant.

		Year		Genotype		Internode		Year x Genotype		Year x Internode		Gen. x Internode		Year x Gen. x Internode	
	df	1		3		1		3		1		3		3	
(a)	Tensile_Strength	**74.9**	***	33.4	***	0.5	ns	12.6	***	2.2	ns	52.2	***	10.7	***
	Modulus	3.2	ns	13.8	***	**107.5**	***	0.4	ns	18.6	***	4.9	**	10.7	***
(b)	IN_Area (mm^2^)	4.8	*	38.4	***	**72.9**	***	2.6	ns	0.7	ns	15.0	***	2.5	ns
	Parenchym (% of Zint area)	0.9	ns	10.3	***	**298.2**	***	2.2	ns	9.1	**	9.5	***	1.7	ns
	EmptySpace_Zint (% of Zint ar.)	3.2	ns	5.2	**	**111.5**	***	1.9	ns	5.5	*	7.9	***	1.6	ns
	Area_Zext (% of section area)	5.2	*	3.4	*	**49.1**	***	1.9	ns	0.0	ns	2.9	ns	4.0	*
	Scl_Zext (% of Zext area)	**23.1**	***	7.6	**	10.0	**	1.2	ns	1.6	ns	1.0	ns	2.3	ns
	VB_density (nb/m^2^ in Zint)	5.6	*	7.4	***	**29.7**	***	5.4	**	0.0	ns	9.2	***	2.4	ns
	VB_number (number)	6.1	*	**53.7**	***	0.1	ns	4.4	**	4.3	*	4.6	**	3.0	*
(c)	ABL (%CW)	4.9	*	9.0	**	**98.1**	***	2.4	ns	1.0	ns	10.3	***	4.1	*
	Yd_ABL	**1062.5**	***	4.0	*	66.9	***	0.9	ns	47.4	***	4.5	*	3.3	ns
	CA (mg/g CW)	17.7	***	**65.6**	***	11.9	**	12.1	***	25.5	***	55.6	***	11.0	***
	FA (mg/g CW)	1.4	ns	101.2	***	**222.8**	***	0.1	ns	4.3	ns	6.2	**	1.5	ns
	Rhamnose (mg/g CW)	**87.0**	***	0.8	ns	49.9	***	3.4	*	1.7	ns	5.6	**	0.5	ns
	H lignin subunit (% of lignin)	11.0	**	3.3	ns	**52.8**	***	1.8	ns	4.9	ns	2.7	ns	2.7	ns
	G lignin subunit (% of lignin)	**88.6**	***	37.5	***	6.3	*	7.1	**	14.3	**	20.3	***	0.1	ns
	S lignin subunit (% of lignin)	**74.9**	***	19.8	***	18.1	***	6.5	**	13.4	**	15.9	***	0.5	ns
	Galactose (mg/g CW)	4.7	*	2.2	ns	**30.9**	***	3.6	*	1.1	ns	3.0	ns	1.0	ns
	GlucHemCel (mg/g CW)	**15.9**	**	0.9	ns	3.0	ns	5.2	**	8.6	**	9.2	***	1.1	ns
	Arabinose (mg/g CW)	0.02	ns	21.2	***	**128.1**	***	5.0	*	2.2	ns	4.6	*	1.8	ns
	XylHemCel (mg/g CW)	7.7	*	16.9	***	**106.6**	***	2.9	ns	0.2	ns	2.1	ns	1.1	ns
	TotHemCel (mg/g CW)	7.2	*	16.0	***	**106.8**	***	3.5	*	0.5	ns	2.9	ns	1.1	ns
	GlucCel (mg/g CW)	**31.8**	***	0.7	ns	15.8	**	0.1	ns	1.2	ns	2.9	ns	0.3	ns
	XylCel (mg/g CW)	0.03	ns	**2.9**	ns	0.1	ns	0.2	ns	0.0	ns	0.3	ns	0.1	ns
	TotCel (mg/g CW)	**23.8**	***	0.4	ns	12.1	**	0.1	ns	0.9	ns	2.2	ns	0.2	ns

*p*-values: ‘***’ < 0.001; ‘**’ < 0.01; ‘*’ < 0.05; ‘ns’ (non significant) > 0.05.

**Table 3:** General mean and coefficients of variation given by the ANOVA for (a) mechanical properties of internode-based biocomposites variables, (b) histological variables, and (c) biochemical variables, measured on 4 genotypes (GIG_B, GOL, FLO, and MAL), for 2 internode positions (bottom and top), in 2 years (2014 and 2015). Means are given per internode in the two last columns and are indicated in bold for significant internode effects.

		Mean	CV (%)	Bot Mean	Top Mean
(a)	Tensile_Strength	36.4	1.3	36.4	36.5
	Modulus	3096.3	3.3	**3202.5**	**2987.6**
(b)	IN_Area (mm^2^)	43.0	25.8	**56.6**	**29.3**
	Parenchym (% of Zint area)	61.3	10.1	**45.9**	**76.7**
	EmptySpace_Zint (% of Zint area)	12.1	46.3	**20.6**	**3.5**
	Area_Zext (% of section area)	21.2	21.9	**25.9**	**16.5**
	Scl_Zext (% of Zext area)	50.5	22.6	**55.8**	**45.3**
	VB_density (nb/m^2^ in Zint)	3.3	18.8	**2.8**	**3.8**
	VB_number (number)	101.3	18.8	102.0	100.5
(c)	ABL (%CW)	23.5	2.9	**24.8**	**22.1**
	Yd_ABL	1039.7	8.2	**1241.3**	**838.0**
	CA (mg/g CW)	18.0	3.4	**18.7**	**17.2**
	FA (mg/g CW)	4.4	5.1	**3.8**	**5.0**
	Rhamnose (mg/g CW)	0.4	12.5	**0.3**	**0.4**
	H lignin subunit (% of lignin)	2.4	16.2	**1.7**	**3.1**
	G lignin subunit (% of lignin)	58.9	1.8	**57.9**	**59.8**
	S lignin subunit (% of lignin)	38.7	3.3	**40.3**	**37.1**
	Galactose (mg/g CW)	2.8	10.6	**2.5**	**3.2**
	GlucHemCel (mg/g CW)	6.0	12.6	5.5	6.5
	Arabinose (mg/g CW)	20.5	6.7	**17.1**	**23.8**
	XylHemCel (mg/g CW)	170.9	6.2	**148.3**	**193.4**
	TotHemCel (mg/g CW)	200.5	6.1	**173.6**	**227.4**
	GlucCel (mg/g CW)	419.5	5.5	**441.0**	**398.0**
	XylCel (mg/g CW)	40.9	12.3	41.3	40.6
	TotCel (mg/g CW)	460.4	5.9	**482.3**	**438.6**

Regarding mechanical properties, the bottom and top internodes yielded different modulus values (3203 and 2988 MPa, respectively), while the main internode effect of tensile strength was not significant.

## Data Availability

The original contributions presented in this study are included in the article. Further inquiries can be directed toward the corresponding author.

## Abbreviations

The following abbreviations are used in this manuscript:

ABL	Lignin content according to acetyl bromide lignin method (% CW)
Area_Zext	Stem section rind fraction, in percentage of the internode section area
Arabinose	Amount of arabinose (mg/g CW)
Blue_Zint	Blue corresponding to less lignified cell wall (% of internal area)
bot	Bottom internodes
CA	Ester-linked p-coumaric acid (mg/g CW)
CW	Cell wall
EmptySpace_Zint	Empty space (% of internal area)
FA	Ferulic acid (mg/g CW)
G	Guaiacyl subunit (% of lignin)
Galactose Gen.	Amount of galactose (mg/g CW) Genotype
GluCell	Amount of glucose from cellulose (mg/g CW)
GluHem	Amount of glucose from hemicelluloses (mg/g CW)
H	p-hydroxyphenyl subunit (% of lignin)
IN_Area Intern.	Internode section area (mm^2^) Internode
Parenchym PCA	Parenchyma tissue (% of internal area) Principal component analysis
S	Syringyl subunit (% of lignin)
Scl_Zext	Sclerenchyma tissue (% of external area)
top	Top internodes
TotHem	Amount of hemicelluloses (mg/g CW)
VB_density	Density of vascular bundles in the internal area (nb/mm^2^)
VB_number	Number of vascular bundles in the internal and central areas
XylHem	Amount of xylose (mg/g CW)
Zext	External area (mg/g CW)
Zint	Internal area (mg/g CW)
